# Bone weathering in a Mediterranean climate region: An experimental case study from Doñana National Park (Spain)

**DOI:** 10.1371/journal.pone.0335508

**Published:** 2025-10-31

**Authors:** Marcos Pizarro-Monzo, Laura Domingo, Juan José Negro, Enrique Cantero, David M. Martín-Perea, M.Soledad Domingo

**Affiliations:** 1 Departamento de Geodinámica, Estratigrafía y Paleontología, Universidad Complutense de Madrid, Madrid, Spain; 2 Earth and Planetary Sciences Department, University of California Santa Cruz, United States of America; 3 Departamento de Ecología Evolutiva, Estación Biológica de Doñana-CSIC, Seville, Spain; 4 Museo Nacional de Ciencias Naturales-CSIC, Madrid, Spain; 5 Institut Català de Paleoecologia Humana i Evolució Social-IPHES, Tarragona, Spain; 6 Instituto de Evolución Humana en África (IDEA), Universidad de Alcalá de Henares, Madrid, Spain; University of Gothenburg: Goteborgs Universitet, SWEDEN

## Abstract

Bone weathering constitutes a highly informative and commonly studied variable in taphonomic analyses. Vertebrate paleontologists, zoo-archeologists and forensic anthropologists have used weathering as a taphonomic clock to ascertain the exposure time of a bone assemblage before burial. Given that climatic conditions largely govern the weathering process, it is essential to investigate the effects of weathering across various climatic settings. This study analyzes the bone weathering process at Doñana National Park (Spain), a Mediterranean climate area. In 2018, we set an experiment in which four bones, three tibias and one skull, belonging to the main ungulates from Doñana (red deer, fallow deer, horse and wild boar) were placed in an enclosure and exposed to the natural environmental conditions. We present here results after almost 6 years of exposure. Over the study period, the most exposed area of the bones reached weathering stages 1 and 2, an intermediate progression between semi-arid tropical savannas, where weathering stages were initially described, and temperate and colder climates. By the final observation, the tibia of the horse, the heaviest taxon in our study, only has reached weathering stage 1, so our study agrees with previous studies in that the rate of weathering differs across body size, being slower in larger animals. The skull of *Sus scrofa* stands out for exhibiting modifications that differ from those observed in the tibias, probably due to the different structural anatomy of this bone. We have characterized the local meteorological conditions throughout the experiment and the soil composition as they might play a role in the weathering progression. This research constitutes a first attempt to calibrate the weathering scale in a Mediterranean climatic context, a setting that contains abundant and important fossil assemblages but that lacks bone weathering calibration.

## Introduction

Defined as the process through which the original microscopic components—organic and inorganic—of a bone are separated or destroyed by physical and chemical agents affecting its surface [1], bone weathering analysis is crucial for understanding the processes that contribute to the formation of fossil assemblages. In her seminal work, A. K. Behrensmeyer (1978) proposed a descriptive categorization of bone weathering based on the systematic observation of modern taphonomic assemblages from Amboseli National Park (Kenya), establishing six stages of modification [1]. Main features for these stages, as originally defined by Behrensmeyer [1], are: 1) Weathering Stage 0 (WS0): bone is fresh with no cracking or flaking; tissues like fat, skin, or marrow may still be present. 2) Weathering Stage 1 (WS1): bone develops fine surface cracks, often along fiber structure, with possible remnants of soft tissue. 3) Weathering Stage 2 (WS2): flaking begins along cracks and might progress until outermost bone is mostly gone. 4) Weathering Stage 3 (WS3): surface becomes rough and fibrous as outer layers are fully removed; bone fibers still tightly bound together; minimal to no soft tissue remains. 5) Weathering Stage 4 (WS4): deep weathering causes coarse texture and splintering, penetrating into inner cavities; splinters might be loose enough to detach from the bone. 6) Weathering Stage 5 (WS5): bone disintegrates and loses its original structure.

Bone weathering is associated with the specific climatic features of the region where bones are found. While early studies emphasized temperature and moisture fluctuations, cumulative research has increasingly pointed to a potentially important role of sunlight and UV radiation [1–4], although further controlled experiments are still needed to confirm this effect. Soil pH levels also affect the weathering process, and both very acidic and alkaline soils can weaken the overall structure of the bone and make it more prone to weathering processes [1,5].

Bones exposed to fewer environmental fluctuations—such as wet–dry, cold–hot, or freeze–thaw cycles—and deposited in shaded, neutral pH soils weather more slowly, as seen in colder temperate, continental, periglacial, or polar climates and densely vegetated tropical rainforests [2,4,6–12], compared with desert or semi-arid tropical environments where drier conditions and prolonged solar radiation accelerate weathering [1,2,13]. In addition to the general climatic conditions of a study area, microhabitat variability and localized environmental factors can lead to differential weathering responses. Variations in ground cover, soil characteristics, and localized temperature and precipitation patterns may significantly influence the weathering process [1]. The intrinsic characteristics of the bone assemblage—such as the skeletal elements present, age of individuals, body size, and taxonomic identity—also influence weathering patterns. In general, denser and stouter skeletal elements belonging to large-sized vertebrates will weather at a slower rate than less dense, slender bones belonging to small-sized vertebrates [1,14–16]. Juvenile bones, which are thinner and porous, exhibit faster weathering rates compared to adult skeletons [17], though additional research is necessary in this regard. Lastly, skeletal differences among different vertebrate classes also translates into different weathering rates so, for example, bird bones tend to weather at a faster rate than mammalian bones [17,18].

The progressive and unidirectional nature of weathering made it feasible to relate weathering stages to the time of exposure of an animal remains before burial [1]. Once burial takes place, weathering does not advance or proceeds at a much slower pace [1]. Weathering is therefore considered a “taphonomic clock” [3] offering a “rough but useful” estimate of exposure duration that other taphonomic variables often cannot provide. When analyzing the fossil record, weathering data serve as an indispensable tool for reconstructing site formation, as the inferred exposure duration, even if it is a rough estimation, provides further, essential information on aspects such as mortality events, burial processes, and depositional environments. (Table 1).

**Table 1:** Predicted relationships between bone weathering patterns in fossiliferous assemblages and site formation scenarios [24–29].

Weathering pattern observed	Interpretation
Early weathering stages	Rapid burial, as exposure to climatic and atmospheric conditions ceases.
Uniform weathering stages	Catastrophic mortality; and, if burial is rapid, bones will show no weathering or only early stages.
Early + uniform weathering stages	Deposition in protected environments leading to retarded weathering (with or without rapid burial), e.g., caves or water bodies.
Diverse weathering stages	Long-term mortality site with attritional accumulation.

The “taphonomic clock” concept has proven highly useful not only to paleontologists and zoo-archaeologists, but also to ecologists and forensic anthropologists (e.g., [10,11,19–23]). Lyman and Fox [14], however, cautioned against assuming a direct correlation between weathering stages and time since death or exposure time, emphasizing that other factors – such as skeletal element type, microenvironmental conditions, and taxon identity – also significantly influence the progression of weathering. They advocated for additional actualistic studies to better understand these dynamics. In the present study, we adopt an actualistic approach to investigate how bone weathering progresses over time in relation to environmental conditions based on a six-year experiment.

The textural features associated with the different weathering stages are generally recognizable across climatic regimes [2,4,8,10,12,13,30]. It is the rate or velocity at which weathering occurs and, therefore, the period at which each of these stages are reached that varies according to the different climatic and environmental features of the studied region [3]. In drawing taphonomical conclusions from the weathering stages of a bone assemblage, it is advisable to calibrate the weathering rates in the study area along with the climatic regime [3]. In this regard, the Global Weathering Project (GWP) -an initiative launched by the Taphonomy Working Group of the ICAZ in 2017 – was designed to compare bone weathering patterns under different climatic regimes worldwide using a simplified version of Behrensmeyer’s protocol, minimizing variables that may affect cross-site comparisons.

The “Mediterranean” climate is characterized by mild, wet winters and hot, dry summers. This setting is typically found on the western coasts of continents, between approximately 30˚ and 40˚ latitude, although the reference region is around the Mediterranean Sea itself [31]. There is debate as to when the onset of Mediterranean-type climate occurred and, while some studies place it at around 3.4 Ma [32,33], other authors propose that the Mediterranean climate may have existed earlier, at least intermittently, during the Neogene or even earlier [34,35].

**Fig 1:**
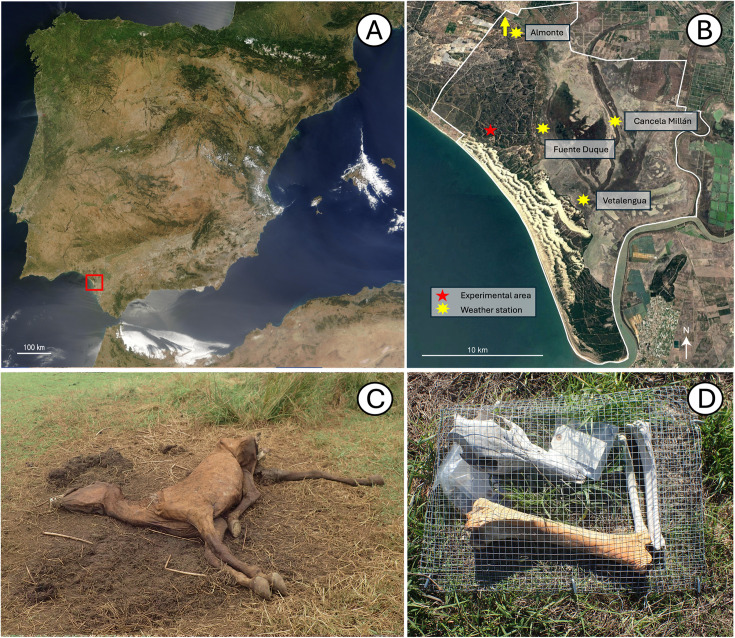
Doñana National Park and the analyzed skeletal material. **(A)** Location of Doñana National Park (Huelva, Spain) on the southwestern coast of Spain. Image courtesy of NASA Visible Earth (public domain image). **(B)** Location of the experimental area and meteorological stations used for this study. The white line marks the boundary of Doñana National Park. The Almonte meteorological station lies outside the map. Image obtained from OrtoPNOA 2019 (scne.es), licensed under CC-BY 4.0. **(C)** Decomposing carcass of *Equus ferus* from which one of the tibiae was obtained (photo taken on September 2017). **(D)** Bones used in this experiment were placed inside a protective metal mesh, with the caudal and ventral facets in contact with the soil and inside a fenced area.

Our weathering experiment is set at Doñana National Park (DNP), characterized by a Mediterranean climate (Figs 1A and 1B) (36.96º N). The average annual rainfall is 580 mm, although there is considerable variation from year to year, ranging from as low as 170 mm in 2004–2005 to as high as 1000 mm in 1995–1996. Precipitation is seasonal, with a wet period from October to April and a dry period from May to September. The average annual temperature is 17°C. Winters are typically mild, with temperatures generally above freezing, while summers are long and hot, with temperatures often exceeding 35°C (>40ºC for some days of July and August).

**Fig 2:**
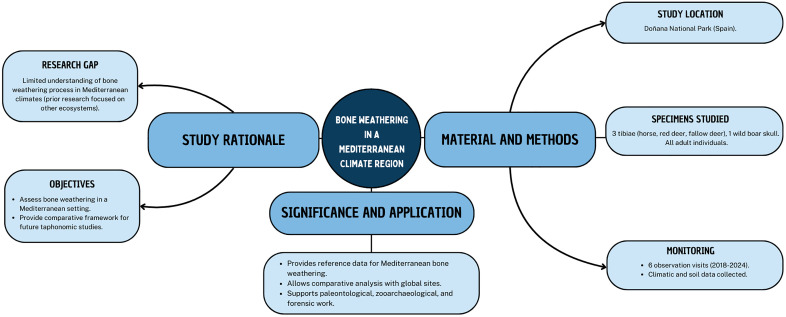
Conceptual diagram summarizing the bone weathering research carried out in the present study.

In the present study, we conducted a taphonomic experiment where a set of bones were exposed to subaerial weathering for 5 years and 4 months (from September 2018 to January 2024). This research will serve as a reference for future taphonomic studies in Mediterranean climatic contexts and enable comparisons with results from other climatic regions (Fig 2). We present the resulting data together with variables recognized as fundamental in the development of bone weathering, such as climate data, soil characteristics, anatomical element, and taxon. This is part of a larger taphonomic monitoring study of the modern vertebrate skeletal assemblages from Doñana National Park, which aims at better understanding the biostratinomic and ecological processes and agents that operate in this ecosystem and how they might impact in the formation of fossiliferous bone assemblages [36].

## Materials and methods

Through this experiment, we monitored four bones under controlled surface conditions over from September 2018 to January 2024. These were: three right tibiae from adult individuals of horse (*Equus ferus)*, red deer (*Cervus elaphus)*, and fallow deer (*Dama dama)*, and a skull from an adult wild boar (*Sus scrofa)*. These are the most abundant feral (horses) and wild mammals at Doñana National Park (Fig 2). We obtained the bones from carcasses found within Doñana National Park (Huelva, Spain). The horse tibia was collected from the lake margin habitat, the red deer tibia from the cork oak woodland, the fallow deer tibia from the marshes, and the wild boar skull from the meadow habitat (refer to [36] for detailed habitat descriptions within Doñana National Park). Ideally, all bones would be sourced from the same habitat to ensure uniform initial micro-environmental conditions; however, this was not feasible under natural conditions, as certain taxa were either absent from some habitats bone assemblages or they did not exhibit WS0 when present. We only know the horse’s approximate death date (around August – September 2017) (Fig 1C). According to their preservation state and presence of soft tissues, the rest of the bones belonged to individuals that died shortly before their date of collection. We retrieved the selected bones from carcasses that retained associated skeletal elements and some articulated portions, indicating a relatively recent time of death. Tibiae were chosen for this long-term weathering study because: (1) they are long bones commonly preserved in faunal assemblages, and (2) its weathering pattern and rate might serve as a model to represent other long bones, which are abundant in skeletons and remains identifiable in the fossil record, even when fragmented. In the case of the wild boar, a skull was used as it came from the inferred most recently deceased adult individual (i.e., WS0, soft tissue and ligaments still present and some skeletal articulation). Scavenging activity had affected the carcass, and only the cranium and uppermost part of the vertebral column were present.

The bones were exposed to subaerial weathering inside a fenced area. We placed them in direct contact with the soil within the experimental area, inside a protective mesh to prevent scavengers and rodents from consuming and dispersing them (Fig 1D). Throughout the experiment, the bones were consistently positioned with the caudal side facing the ground for the tibiae and the ventral side for the skull. The enclosure is within the shrubland habitat of Doñana National Park [36], where the sand dunes are stabilized by vegetation. No shrub or tree was covering or shading the bones (i.e., they were exposed to sunlight). During each field visit, vegetation that could have grown over the previous year was cleared. This vegetation primarily consisted of short grasses and, in most instances, the most exposed surfaces of the bones—namely, the cranial side of the tibiae and the dorsal side of the skull—remained uncovered and visible.

We evaluated periodically the bones in the field and documented them through photography. Bone examinations were conducted on the following dates (approximate time of exposure is indicated in parentheses):

- First record: September 18, 2018 (start of the experiment).
- Second record: January 30, 2019 (~4 months).
- Third record: February 4, 2020 (~1.5 years).
- Fourth record: October 20, 2021 (~3 years).
- Fifth record: December 15, 2022 (~4 years).
- Sixth record: January 26, 2024 (~5.5 years).

As indicated before, the dates of death of the red deer, fallow deer and wild boar are unknown, and we will use the time of exposure indicated above in assessing rates of change in weathering. The horse died in the summer of 2017, and its skeleton was exposed for one year before the tibia was used for this experiment. Therefore, we add one year to the times of exposure indicated above in the analysis of this bone.

We are aware that the protocol for assigning weathering stages to bones is the most advanced stage observed in an area larger than 1 cm^2^ on the whole bone [1]. We conducted a more detailed analysis of the progression of bone weathering, by evaluating different bone sides to determine their weathering stage as follows [37,38]:

- Tibiae: Cranial side, caudal side, medial side, and lateral side.
- Skull: Dorsal side, ventral side, left lateral side, right lateral side.

We performed a descriptive analysis of the weathering stages observed on each bone surface, following the guidelines established by Behrensmeyer [1]. In some cases, we used intermediate states to better reflect what we considered transitional features between stages.

Detailed meteorological data were available for the entire experimental period. Over 1,650,000 meteorological readings were collected, corresponding to temperature (°C), humidity (%), and precipitation (mm), recorded every 5 minutes, along with 1,957 daily solar radiation values (W/m²). We used solar radiation data recorded by the weather station based in the municipality of Almonte managed by the Department of Agriculture, Fisheries, Water, and Rural Development of the Andalusian Government, located approximately 37 km from the experimental area, on the northwestern boundary of the National Park (Fig 1B). The remaining variables were recorded from weather stations located inside the National Park and managed by the Doñana Biological Station (ICTS-RBD). Specifically, the Fuente Duque station, located approximately 5 km from the study area, was the primary reference. In cases where readings were unavailable for certain periods, data from the Vetalengua and Cancela Millán stations, located 12 km and 17 km away, respectively, were used to supplement the missing data (Fig 1B).

This approach allows for the identification of significant meteorological changes, which could have an impact on the rate of progression in the different stages of bone weathering. We calculated daily means as well as daily amplitude averages for temperature (ºC) and humidity (%) and averaged them in monthly data. In the case of precipitation (mm), the total accumulated rainfall per month is provided, as well as the total accumulated rainfall per recording period, to determine the existence of significant differences between visits to the bones. We also estimated daily mean values for solar radiation (W/m^2^) and averaged them to give monthly data. Recording phases for the meteorological data correspond to the periods of time between successive visits to the bones. We performed Kruskal–Wallis tests to compare the distributions of the monthly mean values collected for each recording phase, to determine whether there were significant differences from one recording visit to the bones to the next. Similarly, Mann–Whitney tests were used for pairwise comparisons between each time-successive recording phases.

To evaluate the potential role of edaphic conditions on the bone weathering process, we collected two soil samples from the experimental area, next to the mesh where the bones were placed. We analyzed both samples at the Geological Techniques Unit (Complutense University of Madrid, Spain) to characterize the soil in terms of its granulometry, mineralogy, pH and conductivity. Granulometric analyses were conducted on a sieve stack for particles ranging from 16 mm to 700 µm. For particles ranging from 700 to 0.10 µm, a laser granulometric analysis was performed on a Honeywell Microtrac X100 equipment. X-ray diffraction analyses (powder and oriented aggregates) were performed on a Brucker model D8 ADVANCE diffractometer. We followed USDA guidelines to describe the soil samples.

## Results

### Bone surface analysis

**Fig 3:**
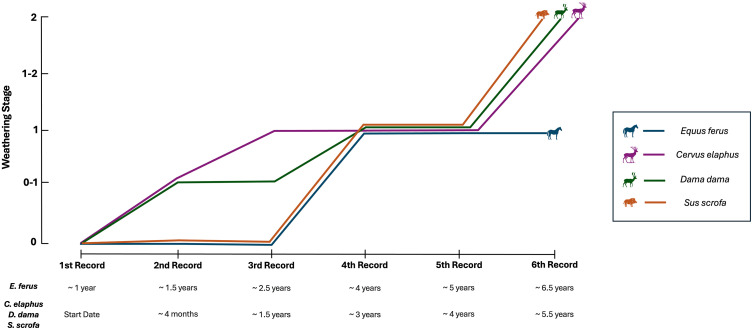
Progression of bone weathering for the different taxa and anatomical elements over the 6 years of experimentation. The maximum weathering stage is shown at each record. Time of exposure is indicated below each record. To ease the reading of the figure the lines have been slightly separated.

The results obtained from the weathering analyses of the bones are summarized in Table 2 and Fig 3. Below, we provide a detailed description of the weathering progression for each element.

**Table 2:** Weathering stages observed for each taxon, anatomical element and bone side. Grayscale shading corresponds to the weathering stage observed, ranging from no shading (WS0) to the darkest shade corresponding to the most advanced stage recorded to date in this study (WS2). Time is shown in approximate years since the start of the study. “d” = weathering timing delayed with respect to Amboseli [3].

		1st Record (~1 year)	2nd Record (~1.5 years)	3rd Record (~2.5 years)	4th Record (~4 years)	5th Record (~5 years)	6th Record (~6.5 years)
*Equus ferus*	Cranial (Exposed)	0	0 (d)	0 (d)	0 (d)	1 (d)	1 (d)
	Lateral	0	0 (d)	0 (d)	0-1 (d)	1 (d)	1 (d)
	Medial	0	0 (d)	0 (d)	1 (d)	1 (d)	1 (d)
	Caudal	0	0 (d)	0 (d)	0 (d)	0 (d)	0-1 (d)
		**1st Record (Start date)**	**2nd Record (~4 months)**	**3rd Record (~1.5 years)**	**4th Record (~3 years)**	**5th Record (~4 years)**	**6th Record (~5.5 years)**
*Cervus elaphus*	Cranial (Exposed)	0	0	0-1	1	1 (d)	2 (d)
	Lateral	0	0	0 (d)	1	1 (d)	1 (d)
	Medial	0	0-1	1	1	1 (d)	1-2 (d)
	Caudal	0	0	0 (d)	0 (d)	0 (d)	0 (d)
*Dama dama*	Cranial (Exposed)	0	0	0 (d)	1	1 (d)	2 (d)
	Lateral	0	0-1	0-1	1	1 (d)	1-2 (d)
	Medial	0	0-1	0-1	1	1 (d)	1-2 (d)
	Caudal	0	0	0 (d)	0 (d)	0 (d)	0-1 (d)
*Sus scrofa*	Dorsal (Exposed)	0	0	1	1	1-2 (d)	2 (d)
	Ventral	0	0	0 (d)	0 (d)	0 (d)	1 (d)
	Right Lateral	0	0	0 (d)		1 (d)	1-2 (d)
	Left Lateral	0	0	0 (d)		1-2 (d)	2 (d)

### *Equus ferus* tibia

**Fig 4:**
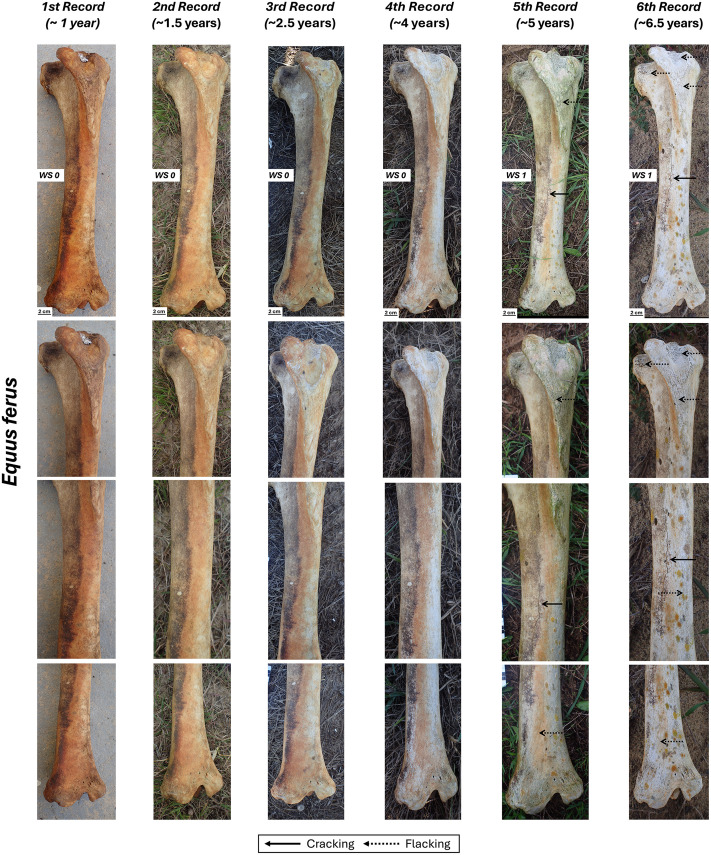
Right tibia of *Equus ferus* in cranial view with its different records and close-up views.

Belonging to a carcass originally located at the margin of a small lake (Fig 1C), the tibia initially displayed a reddish coloration that bleached over time. This bone showed the least amount of alteration, reaching only WS1 after more than six years of exposure (Fig 4, Table 2). The sides exposed to atmospheric conditions showed signs of alteration starting from the fourth record (~ 4 years), in the form of longitudinal cracking following the collagen lines of the cortical bone (Fig 4; S1–S3 Figs). It is worth highlighting that on the medial surface of the tibia, between the fourth and fifth records, there is an apparent reversal of WS1. The fifth record coincided with a period of rainfall and high humidity in Doñana National Park, as also demonstrated by the generalized greenish coloration over the bone surface (probably due to green algae). It is plausible that, under humid conditions, the bone undergoes some expansion that attenuates the cracking typical of WS1. Regarding the caudal side in contact with the soil, we did not observed alterations other than a small fracture on the diaphyseal region. However, this could only be considered a very incipient WS1. (Table 2, S1 Fig). This side of the bone maintains its original reddish color.

### *Cervus elaphus* tibia

**Fig 5:**
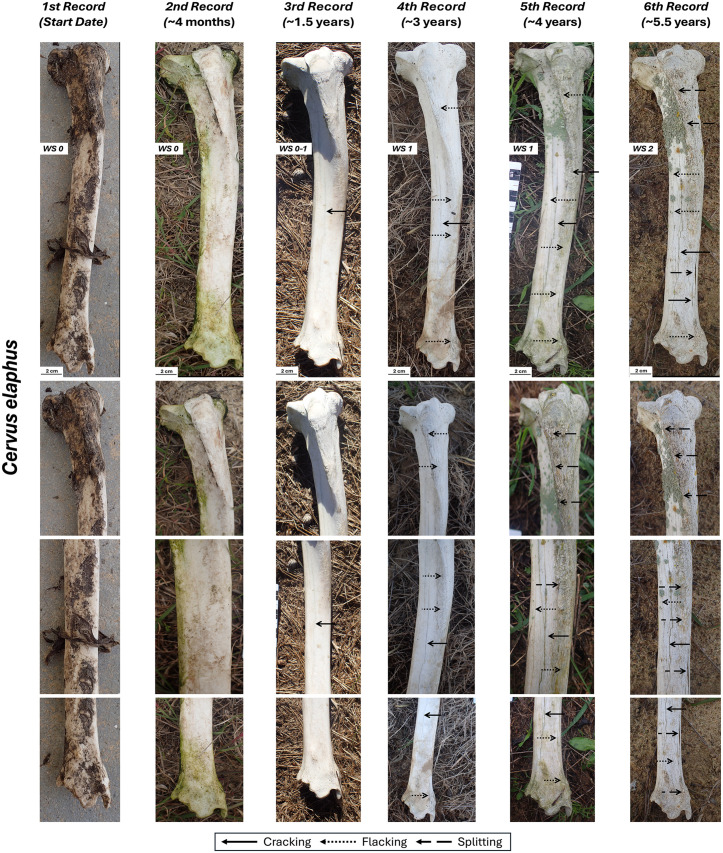
Right tibia of *Cervus elaphus* in cranial view with its different records and close-up views.

When collected, this bone was still partially covered by remains of ligaments, skin, and periosteum over its entire surface, with a higher concentration in the articular surfaces and insertion crests of the proximal epiphysis. On the cranial, medial and lateral sides, the complete loss of these organic remains occurred between the first record (start of the experiment) and the second record (less than 134 days) (Fig 5), possibly due to microbial decomposition processes. Subtle cracks following the degradation of collagen fibers (corresponding to an incipient WS1) were observed on the diaphyseal region of the medial side only 4 months after exposure, although they became more evident in the following record, and they also appeared on the cranial side (Fig 5, Table 2, S6 Fig.). These progressed throughout the experiment, becoming widespread and reaching an initial WS2 in the final record (~ 5.5 years) on the cranial and medial sides of the bone. At this stage, there were not only fractures and cracks, but also cortical exfoliation and splitting, particularly evident as small-scale flaking on the tibial tuberosity, mid-shaft and distal epiphysis and as a longer flake towards the mid-distal diaphysis. The caudal part of the bone (i.e., the downward-facing side) showed no signs of weathering and, even after almost six years of exposure, some remnants of the soft tissue attachments are still present (Table 2, S4 Fig).

### *Dama dama* tibia

**Fig 6:**
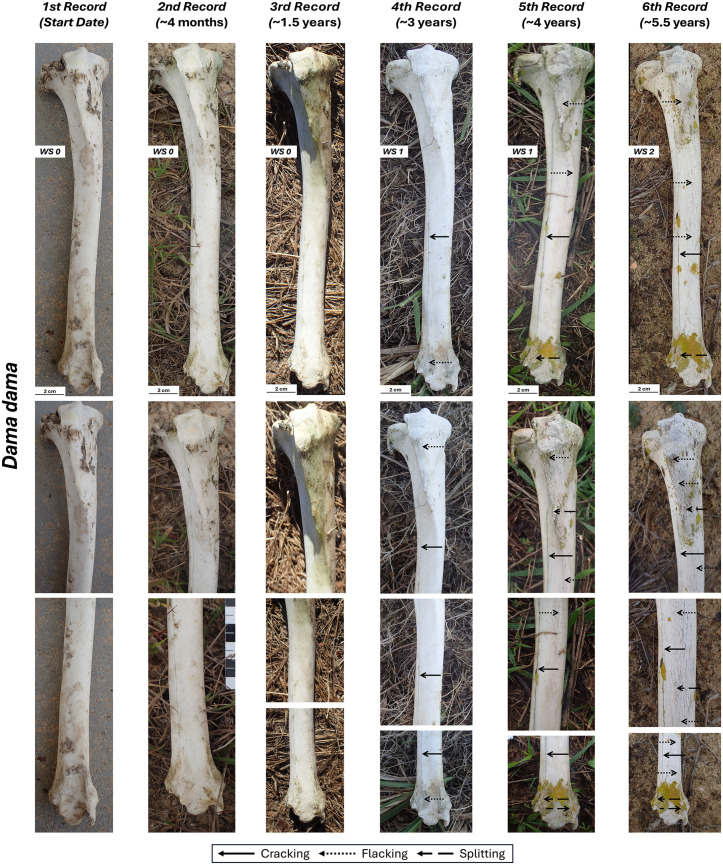
Right tibia of *Dama dama* in cranial view with its different records and close-up views.

This bone followed a similar weathering pattern to the *Cervus elaphus* tibia (Fig 6, Table 2). The only exception was observed on the caudal side where slight cortical fractures, following the degradation of the collagen fibers, are indicative of an early stage 1 in the final record (~ 5.5 years) (S7 Fig). The cranial, lateral, and medial side reached WS1 starting from the fourth record (~ 3 years), although on the lateral and medial views single longitudinal cracks are observed in earlier records and they could correspond to incipient WS1 (Fig 6, S8 Fig, S9 Fig). In the final record (~ 5.5 years), early signs of cortical splitting, characteristic of WS2, were observed on the mid-distal shaft for the cranial and medial view (Table 2).

### *Sus scrofa* skull

**Fig 7:**
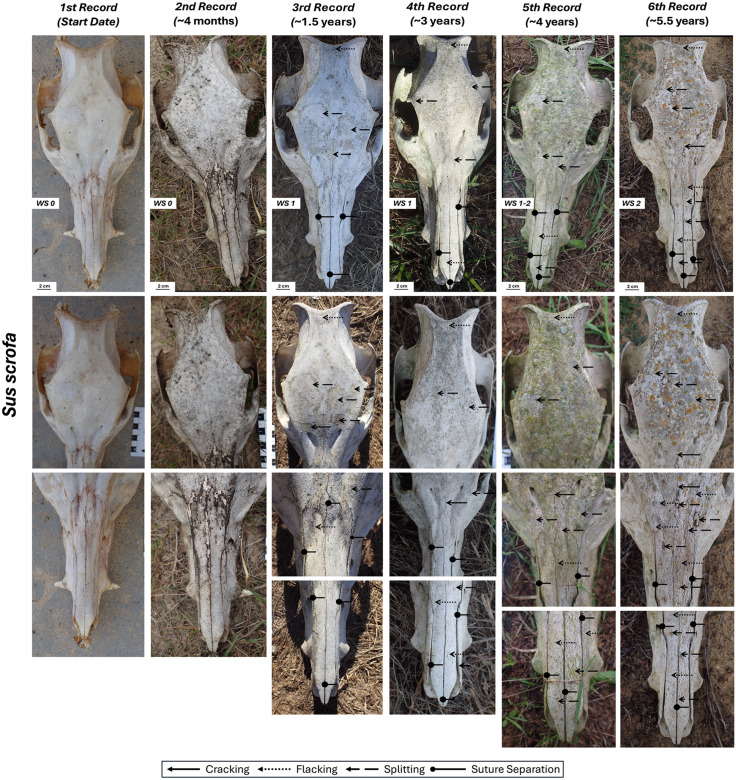
Skull of *Sus scrofa* in dorsal view with its different records and close-up views.

We collected this skull with remnants of ligaments, skin, fat, and periosteum on the occipital, zygomatic arches, nasal and palatal sutures, as well as the alveolar region. The atlas was still connected to the skull. It is complicated to describe weathering stages in this bone as they seem to be characterized by different features than those described by Behrensmeyer [1]. For example, flaking and even some loss of the external part of the bone seems to occur before cracking. In dorsal view, the third record displays these features that we have here classified as WS1 as they are the first bone modification to occur (Fig 7, Table 2). In the second record, the growth of dark-colored fungi along the fronto-occipital and fronto-nasal sutures is observed but no signs of weathering are evident. WS2 starts to become more evident in the fifth and sixth records as flaking and splitting becomes pervasive. This is mostly true for the nasal area as the frontal and occipital areas are covered by lichens. By the fourth record, weathering causes the separation of the nasal sutures.

In the lateral views, weathering modification is again mainly characterized by flaking of the cortical bone since the early stages rather than the typical initial cracking (S10 Fig, S11 Fig). Both the right and left sides show no weathering signs for 1.5 years. The record of 2021 is missing but, after this, it can be observed that the left side has weathered more than the right side so, in the sixth and last record, pervasive exfoliation and flaking are evident on the left side (WS2) and present, but not so widespread, on the right side (WS1–2). Although the cause of this asymmetry remains uncertain, it is possible that the differential weathering observed between the left and right sides of the skull is influenced by orientation to solar exposure during peak hours or partial shielding from adjacent bones (especially the horse tibia) within the protective mesh (Fig 1D).

As for the ventral side of the skull (in contact with the ground throughout the experiment), there is a mosaic-pattern texture located on the retromolar, and palatal regions present since the third record (1.5 years). A close-up view of this region reveals the presence of what seems filamentous structures that might correspond to fungi hyphae, so we tentatively assign this mosaic pattern bone modification to the action of fungi and not to weathering (S13 Fig). This ventral region of the skull reached WS1 in the final record showing some cracking in the palate region (S12 Fig).

Other modifications were documented on this skull throughout the experiment. The first was the progressive loss (detachment) of teeth due to dehydration and the opening of the alveolar region following this sequence: second record, loss of the right canine and left central incisor; third record, loss of the left canine and right first premolar; fourth record, loss of the left first premolar, right second premolar, and right first molar; fifth record, loss of the left second premolar. The second alteration was the progressive separation of the bone’s natural sutures, particularly evident on the palatal region (Fig 7, S12 Fig). Finally, after approximately three years of exposure, the atlas became disarticulated (between 2020 and 2021).

### Climatic variables analysis

**Fig 8:**
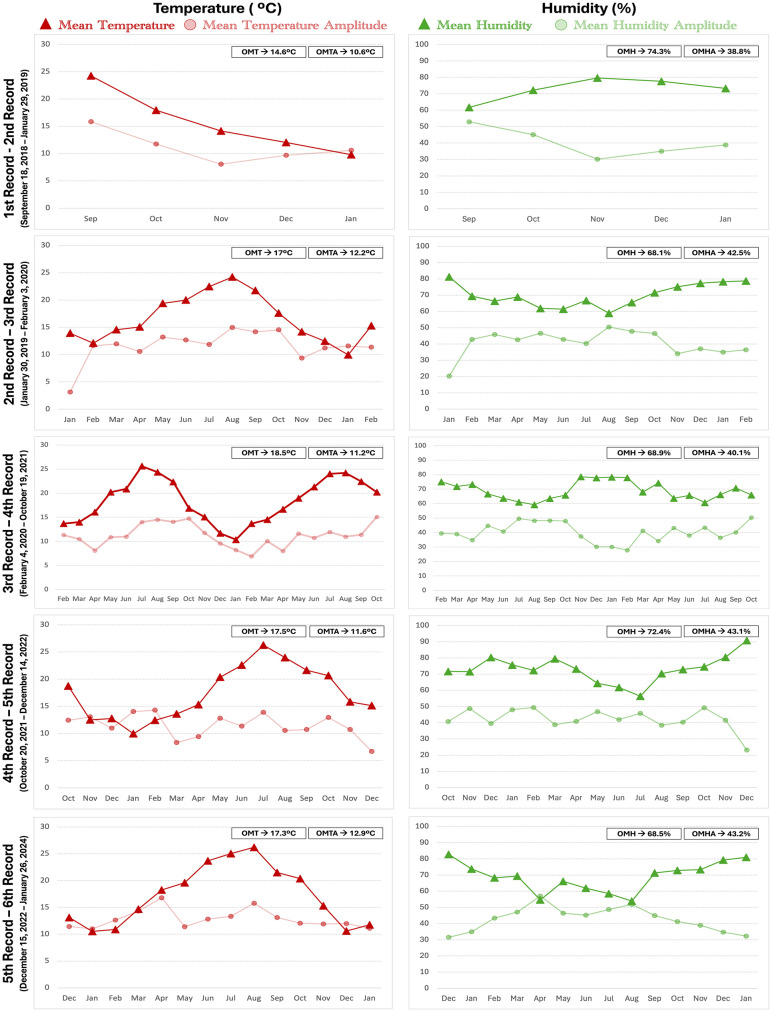
Climatic variables analysis. Left: Temperature (ºC). Right: Humidity (%). OMT = Overall Mean Temperature. OMTA = Overall Mean Temperature Amplitude. OMH = Overall Mean Humidity. OMHA = Overall Mean Humidity Amplitude.

**Fig 9:**
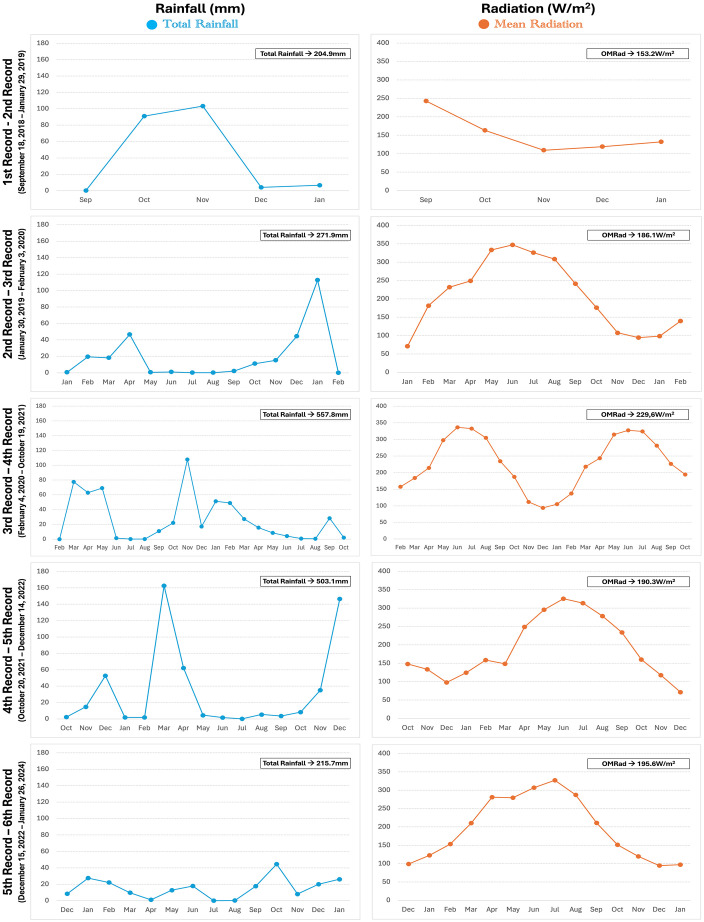
Climatic variables analysis. Left: Rainfall (mm). Right: Solar radiation (W/m^2^). OMRad = Overall Mean Radiation.

The data obtained from the climatic records reflected the characteristics typical of the Mediterranean environment in which Doñana National Park is located. All variables exhibited seasonal patterns (Figs 8 and 9) with temperatures and solar radiation being higher and humidity being lower in the summer months (June to September) and rainfall showing more variability but occurring in spring, autumn and winter. Over the last analyzed years (2021–2024), there has been a general reduction in rainfall. The most recent records showed a clear trend toward drier conditions, with progressively lower precipitation levels. As for the oscillations in temperature and humidity, mean thermal amplitudes are around 11ºC and mean humidity amplitudes go from 38.8% to 43.2% (Fig 8). The climatic data, grouped in periods of time between successive bone examinations, were statistically compared. Although descriptive data show some apparent visual fluctuations in the climatic variables across the study period, Kruskal-Wallis and Mann-Whitney tests showed no statistically significant differences between successive recording periods (S1 and S2 Tables).

### Soil analysis

To better understand if local edaphic conditions might influence bone weathering patterns in Doñana National Park environment, we conducted soil analysis to characterize it in terms of its granulometry, mineral composition and pH. The granulometric analysis concluded that the soil texture was sandy (95.2%–98.4%) with a minimal silt fraction (4.8%–1.6%) for the two soil samples. Potentiometric and conductometric analyses of the soil samples indicated a moderately alkaline pH (pH = 8.1 ± 0.1–7.9 ± 0.1) with non-saline conductivity values (57 ± 6–62 ± 6 µS/cm).

X-ray powder diffraction showed that the four crystalline phases present in the samples were: quartz (86–87%), phyllosilicates (6%), potassium feldspar (5–4%), and plagioclase (3%). The oriented-aggregate X-ray diffraction indicated that the clay minerals in the samples were mica-illite (78%), chlorite (11%), and kaolinite (11%).

## Discussion

We present here the results from the initial phase of a long-term weathering experiment performed on a set of bones exposed for almost 6 years in the Mediterranean climatic context of Doñana National Park, providing a baseline for ongoing research. To calibrate a complete weathering scale in this climatic context, this experiment is intended to continue long term (>15 years). After years of investigation by numerous researchers in varied regions of the world, it is evident that there is a direct relationship between weathering progression and climatic/meteorological conditions and oscillations (temperature, humidity, precipitation, solar radiation) so, together with the weathering results, we provide here a characterization of the climatic variables during the time that this experiment has been running so far, as well as a description of the soil features.

When comparing weathering progression with Behrensmeyer’s work [1] and other research on bone weathering [2,6,8,12,13,15,30,39,40], pattern and rate must be accounted for. In the case of the three tibiae analyzed here, the pattern or the textural features that characterize the early weathering stages are similar to those described by Behrensmeyer [1], i.e., longitudinal cracking for WS1 that advances to flaking for WS2.

The wild boar skull has also weathered through time, but the pattern differs from the original definition of each of the stages. For example, in this bone flaking occurs before cracking (Fig 7, S10–S12 Figs). Other authors also have observed differential weathering features in skulls. Gutiérrez et al. [30] conducted a weathering experiment on modern guanaco skeletons of different ages in the Olavarría party (Buenos Aires province, Argentina). For the adult skull, they observed desiccation with abundant exfoliation and flaking and, in their case, the rate of weathering in skulls was faster compared to other skeletal elements to the point that by the seventh year of observation portions of the frontal and nasal bones were disintegrating. Behrensmeyer [1] also indicated that some bones exhibit different weathering patterns and, although she was not explicitly referring to skulls, she noted cases of extensive flaking without significant cracking. Long bones, such as tibias, have fundamentally an endochondral osseous origin, whereas skulls contain skeletal elements of dermal origin [41]. This differing osseous nature most probably plays a role in the dissimilar weathering patterns observed between these two skeletal elements.

As for the rate of weathering, overall, the bones from Doñana National Park display a slower progression of the weathering stages compared to Amboseli National Park skeletal remains (Tables 2 and 3). This slower weathering rate is accentuated in the case of the horse tibia, whose exposed area (cranial side) shows WS1 after 6.5 years of exposure. Weathering rates for the red deer and fallow deer tibias are very similar: their exposed areas display WS1 between 1.5 to 4 years and, by 5.5 years, they have reached WS2. There is some overlap between these rates and Amboseli rates, but they clearly fall in the slower end of Amboseli range for these stages [1], which is more evident if the Behrensmeyer and Miller [3] reference is considered.

**Table 3:** Time elapsed to reach early weathering stages (WS1 and WS2) in this and other neo-taphonomic studies.

Environment	Weathering Stage		Global Horizontal Irradiation (kWh/m^2^ per year) [42]	Reference
	WS1	WS2		
**Savanna**				
Amboseli National Park, Kenya	0-3 years	2-6 years	2167.8	[1]
Tsavo National Park, Kenya	8 months-2 years	1.5-5 years	2017.6	[39]
Virunga National Park, Dem. Rep. Congo	0-2 years	2-4 years	1871.7	[2]
**Desert**				
Abu Dhabi Desert, UAE	0-10 years	10-15 + years	2222.7	[15]
Wadi Enoqiyya, Jordan Desert, Jordan	Less 6 weeks	1-3 years	2152.1	[13]
**Tropical Rainforest**				
Ituri Rain Forest, Dem. Rep. Congo	15 + years	15 + years	1901.6	[2]
**Temperate Grassland and Forests**				
Draycott, UK	8 + years	N/A	1043.6	[6]
Neuadd, Wales, UK	22 + years	N/A	973.4	[8]
Riofrío, Spain	4 + years	16 + years	1685.9	[40]
Olavarría, Argentina	0-5 years	2-8 + years	1730.3	[30]
Cantabria, Spain	4 + years	N/A	1250.4	[12]
Yellowstone National Park, USA	1-6 years	6-10 years	1513.4	[10]
**Mediterranean**				
Doñana National Park, Spain	4 months-6.5 + years	4 + years	1882.4	This study

The wild boar skull reaches a transitional WS1–2 at 4 years and WS2 at 5.5 years. Again, this corresponds to an overlapping, but delayed rate compared to Amboseli material.

The medial and lateral sides of the three tibias analyzed started to weather before the most exposed side (cranial side). In all the cases, this initial weathering corresponds to single longitudinal cracks. We are not sure why weathering starts on these lateral and medial regions and could be related to the disposition and distribution of the collagen fibers but by the end of the observations the cranial, most exposed region presents the most advanced weathering stage.

The delayed weathering rate of the horse tibia compared to the red deer and fallow deer tibias might respond to the different body sizes of these animals. Horses are animals with a body mass of >300 kg, whereas red deer body mass is around 100–150 kg and fallow deer ranges from 28 to 63 kg. Our results agree with previous studies where it has been observed that weathering is slower in the skeletal elements of larger, heavier animals than in smaller ones [1,3]. However, in addition to body mass, differences in bone mineral density may also influence weathering resistance. Denser cortical bone, which is often associated with larger taxa, may be more resistant to early stages of weathering processes such as cracking and flaking [1,41,43]. Other bone characteristics associated with body size or taxonomic affiliation—such as cortical thickness, bone microstructure, and marrow cavity size—likely influence the progression of weathering. However, further experimental research is required to clarify the relationship between these features and the weathering process.

Non-exposed areas of the bones (those in contact with the ground) were unweathered (WS0) or slightly weathered (WS1) by the final observation. This pattern, consistent with Behrensmeyer [1], suggests the importance of sunlight exposure in weathering, alongside other potential contributing factors such as increased moisture retention and microbial processes on the down-facing surfaces.

Results from the soil analyses revealed their sandy nature (95.2–98.4% sand). Sandy soils have a high drainage capacity, which promotes surface dehydration due to its low moisture retention capacity [20]. This may accelerate the physical and chemical erosion of bone structures, thus favoring the weathering process compared to environments with soils that retain moisture. However, the high porosity of sandy soils may also allow upward water movement through capillary action and evaporation, potentially maintaining a more stable moisture level beneath the bones. This dual effect could significantly influence weathering dynamics, underscoring the need for further experimental investigations on the influence of soil types on bone weathering.

In terms of the pH or salinity, the soil in the study area is moderately alkaline and non-saline. These characteristics suggest that the soil is not particularly reactive in ways that would enhance weathering bones. Previous research has shown that both highly acidic and highly alkaline soils can accelerate bone degradation by altering the hydroxyapatite structure and facilizing collagen breakdown [1]. Soils with high salinity may promote chemical weathering through salt crystallization processes. To date, however, little research has addressed this issue, and as indicated before, further studies are needed to ascertain the role of soil characteristics in bone weathering.

From the results obtained here, and comparing with studies carried out in different regions and climatic contexts (Table 3), we suggest that under Mediterranean conditions bone weathers at a pace that is intermediate between those observed in temperate regions (grasslands and mixed forests) such as Neuadd, Draycott or the Cantabrian region of Spain [6,8,12] and those observed in tropical savannas of Amboseli National Park and Virunga National Park [1,2].

The climatic data analyzed here (Figs 8 and 9) conforms to the *Csa* climate in the Köppen climate classification that defines hot-summer Mediterranean climates. *Csa* climates are seasonal both in terms of the temperature and the precipitations, whereas the seasonality of *Aw* climates from tropical dry savannas mainly comes from the precipitation. In *Aw* climates, temperatures are higher and stable throughout the year [44]. The autumn, winter and spring months’ conditions at Doñana, being colder and more humid than at tropical savannas, might slow down weathering. Temperate regions, and specifically *Cfb* climates like those of the regions mentioned above, do not have a dry season and remain humid and cold/cool throughout the year, conditions that would be expected to slow down the weathering process even more. At Doñana, summer temperatures are high (often reaching extreme values), and precipitations are tiny (Figs 8 and 9) so bones can become very dry, conditions similar to tropical savannas and significantly different to temperate regions. To evaluate the frequency of thermal stress and its potential influence on bone weathering, we classified each day of the experimental period (n = 1956) according to mean daily temperature: “Extreme heat” (≥30ºC), “Hot” (25–29.9ºC), “Moderate” (15–24.9ºC), and “Cool/Cold” (<15ºC). This classification reflects ecologically meaningful threshold in Mediterranean environments, where high daily means are associated with increased desiccation stress of exposed biological remains and intense solar exposure. Based on this scheme, we recorded 4 extreme heat days (0.2%), 153 hot days (7.8%), 1060 moderate days (54.2%), and 739 cold days (37.8%), thought the study period. These data provide environmental context for interpreting weathering progression in the analyzed specimens.

Solar radiation is also proposed as a major weathering driver and is highly dependent on latitude (apart from other variables such as cloud coverage) [1–4,15,22]. For comparative purposes, instead of using the radiation data coming from the Almonte station of our study, we compiled data from the free web “Global Solar Atlas 2.0” [42] (Table 3). This online tool provides different measures of solar data to obtain solar power data across the world. One of the measures is the Global Horizontal Irradiance (GHI), which estimates the total irradiance from the Sun on a horizontal surface on Earth. Yearly GHI for Table 3 locations, such as for example Neuadd, Cueto in the Cantabria Region, Doñana National Park and Amboseli National Park, are 973.4 kWh/m^2^, 1250.4 kWh/m^2^, 1882.4 kWh/m^2^ and 2167.8 kWh/m^2^, respectively. This shows the expected increase in solar radiation as a region is closer to the Equator and a likely correlation with weathering rates.

We gathered climatic data not only to characterize our study area environmentally but also to evaluate whether there were differences among the periods in between visits. No significant differences were observed so climatic conditions have been similar and stable throughout the time evaluated.

Finally, we are aware of the limitations of our study with respect to the small sample size. Different skeletal elements might show some differences in the pattern and weathering rate as well, so the use of complete skeletons would be advisable in future experiments. Also, we are carrying out our study on a clearing of the shrubland habitat within Doñana National Park, but there are other habitats (marshland, swamp, woodland) whose (micro)environmental conditions might produce differences in the progression of weathering. Ideally, experiments with complete skeletons of different taxa in different habitats should be conducted, and we aim to address these concerns in the future. However, we must also consider that this is a naturally protected area and installation of enclosures is not allowed throughout the park. Notwithstanding these recommendations and future experimental refinements, our study constitutes an important baseline contribution to the investigation of bone weathering in a Mediterranean climatic context.

## Conclusion

We present the results of a bone weathering experiment in the Mediterranean climatic setting of Doñana National Park. Four bones (three tibias and one skull) of the main feral and wild ungulates from Doñana were exposed for almost six years to natural environmental conditions. By the final observation, bones reached WS1 or WS2. This weathering progression is slower than that observed in tropical savannas and faster than observed in colder temperate regions.

Non-exposed bone sides do not weather or weather very slowly, and bones of larger animals (in our case, the horse) weather at a slower pace than bones of smaller animals (red deer and fallow deer). These observations agree with previous research by other authors.

The wild boar skull analyzed here weathers through time but shows different textural patterns than those observed in long bones. In the skull, flaking and exfoliation occur earlier and are more prevalent than cracking. The different osteological architecture and origin of the skull bones compared to long bones might explain the disparity in weathering patterns.

This is intended to be a long-term project, so future observations will allow us to investigate the progression into more advanced weathering stages and complete the weathering scale calibration in a Mediterranean context. Bone weathering is a critical variable in taphonomic analyses, providing valuable insights into the pre-burial history of skeletal remains. Fossil sites formed under the Mediterranean climatic context are abundant, and we hope this study serves as a reference for taphonomists working in this specific setting.

## Supporting information

S1 Fig Right tibia of *Equus ferus* in caudal view with its different records and close-up views. (TIFF)

S2 Fig Right tibia of *Equus ferus* in lateral view with its different records and close-up views. (TIFF)

S3 Fig Right tibia of *Equus ferus* in medial view with its different records and close-up views. (TIFF)

S4 Fig Right tibia of *Cervus elaphus* in caudal view with its different records and close-up views. (TIFF)

S5 Fig Right tibia of *Cervus elaphus* in lateral view with its different records and close-up views. (TIFF)

S6 Fig Right tibia of *Cervus elaphus* in medial view with its different records and close-up views. (TIFF)

S7 Fig Right tibia of *Dama dama* in caudal view with its different records and close-up views. (TIFF)

S8 Fig Right tibia of *Dama dama* in lateral view with its different records and close-up views. (TIFF)

S9 Fig Right tibia of *Cervus elaphus* in medial view with its different records and close-up views. (TIFF)

S10 Fig Skull of *Sus scrofa* in left lateral view with its different records and close-up views. (TIFF)

S11 Fig Skull of *Sus scrofa* in right lateral view with its different records and close-up views. (TIFF)

S12 Fig Skull of *Sus scrofa* in ventral view with its different records and close-up views. (TIFF)

S13 Fig Skull of Sus scrofa in ventral view. Close-up of the palatal region with fungi hyphae filamentous presence.
(TIFF)

S1 Table Results of the Kruskal-Wallis test on climatic variables across all recording phases. (PDF)

S2 Table Results of the Mann-Whitney tests (p-values are shown) comparing climatic variables between time-successive recording phases. (PDF)
