# Supplementary figures and images for: Bone weathering in a Mediterranean climate region: An experimental case study from Doñana National Park (Spain)
Source: PLoS One. 2025 Oct 31;20(10):e0335508. doi: 10.1371/journal.pone.0335508 (PMC12578198; doi:10.1371/journal.pone.0335508)

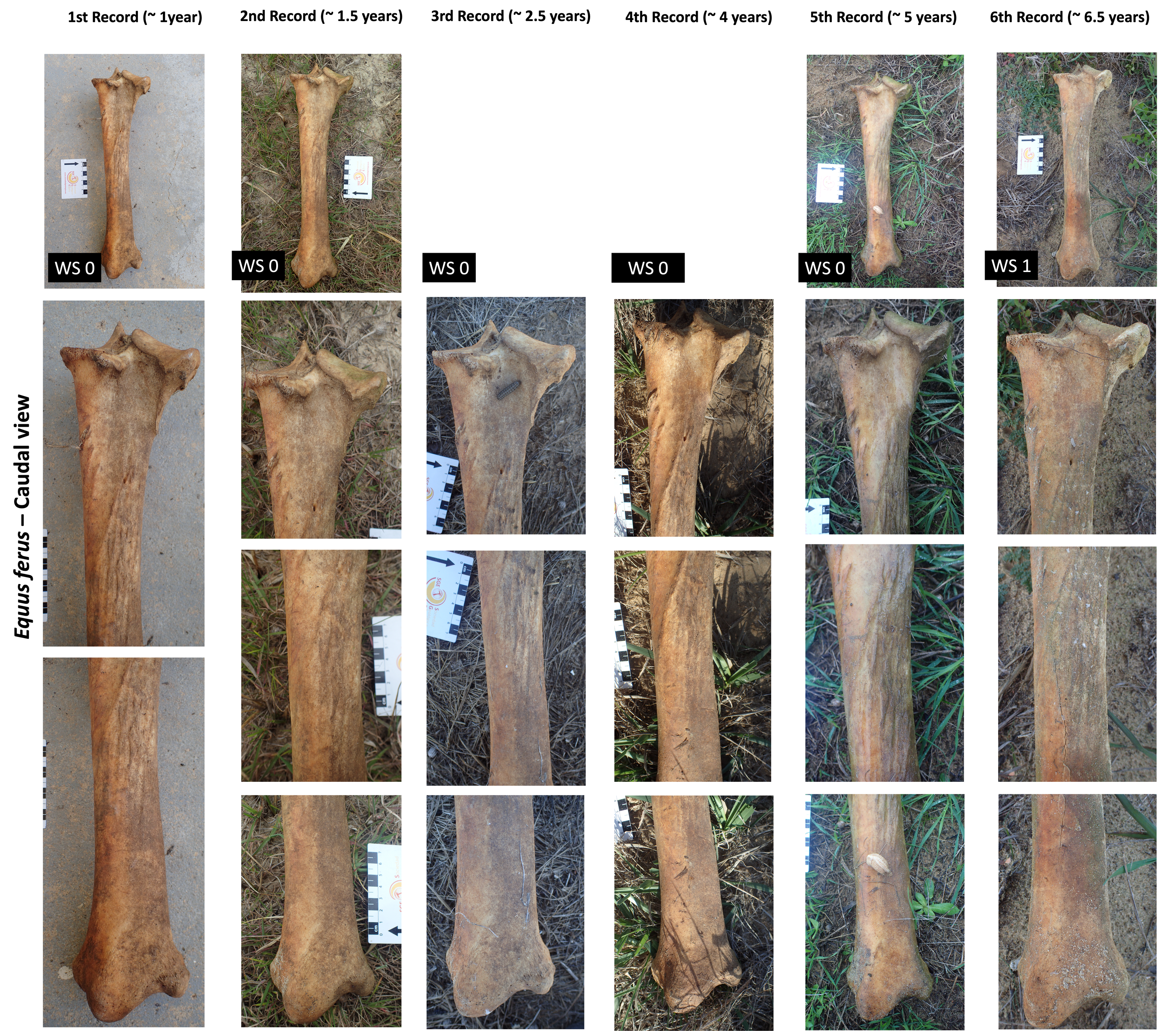

Supplement: S1 Fig — (TIFF) [file pone.0335508.s001.tiff]

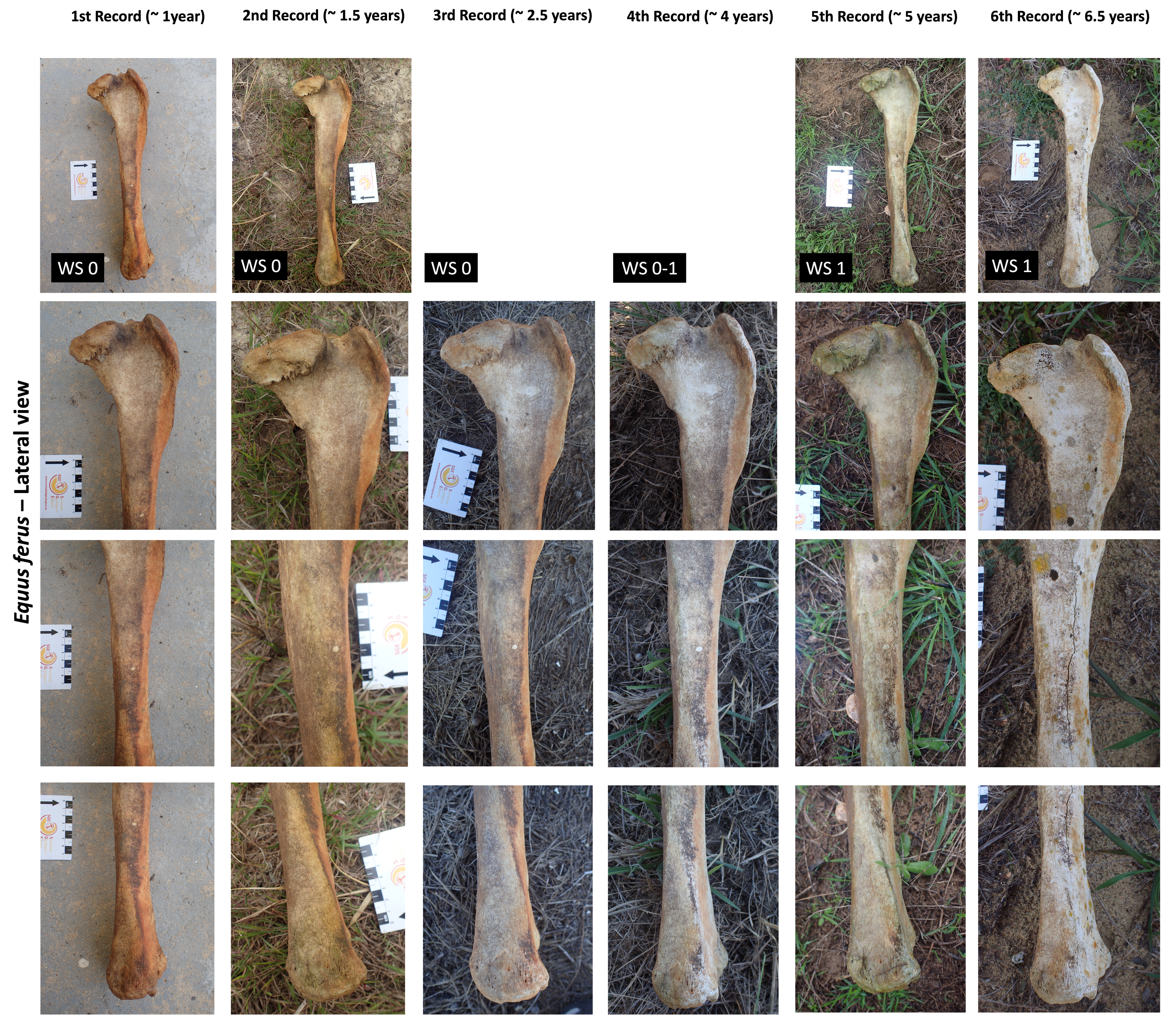

Supplement: S2 Fig — (TIFF) [file pone.0335508.s002.tiff]

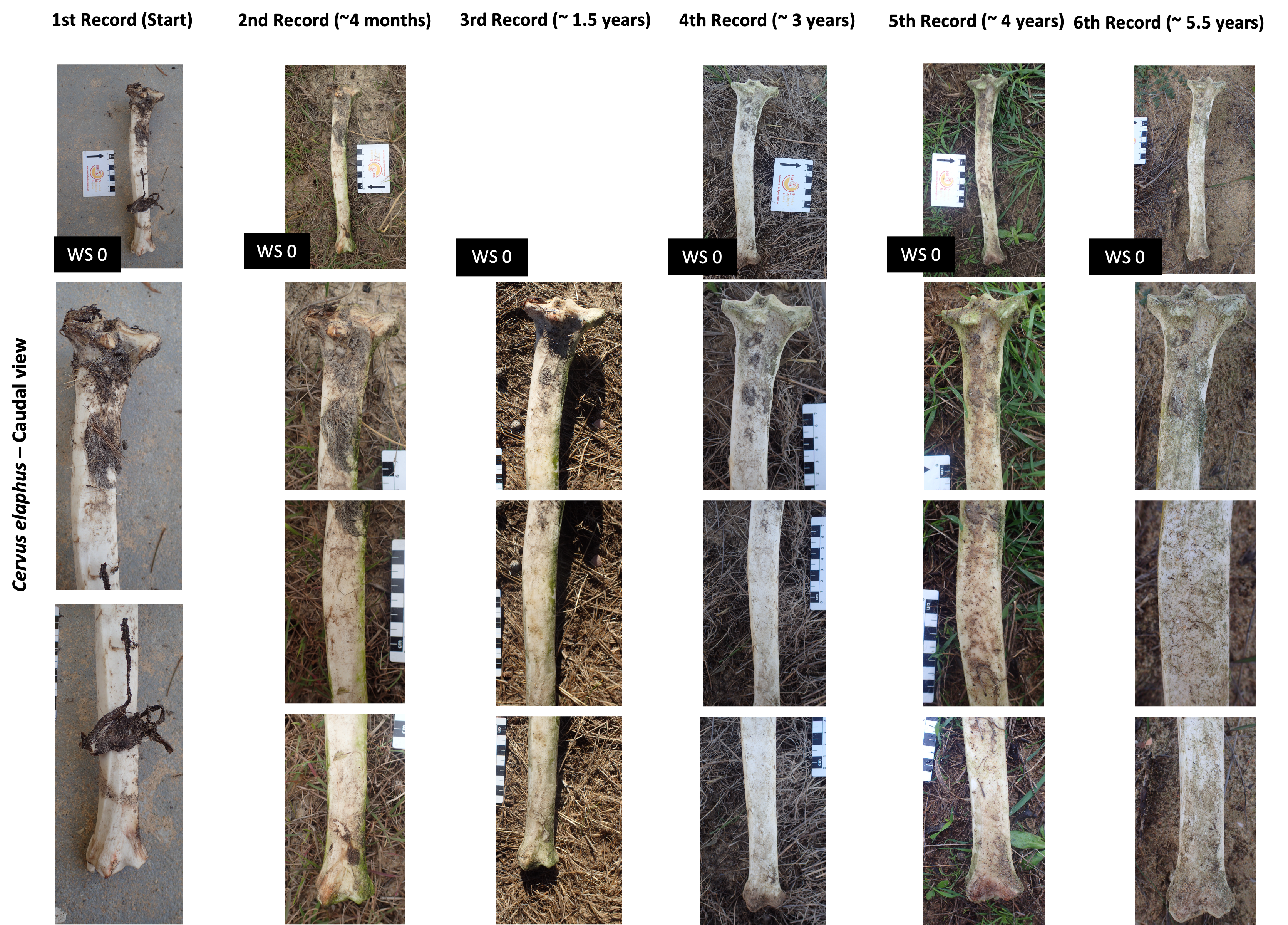

Supplement: S4 Fig — (TIFF) [file pone.0335508.s004.tiff]

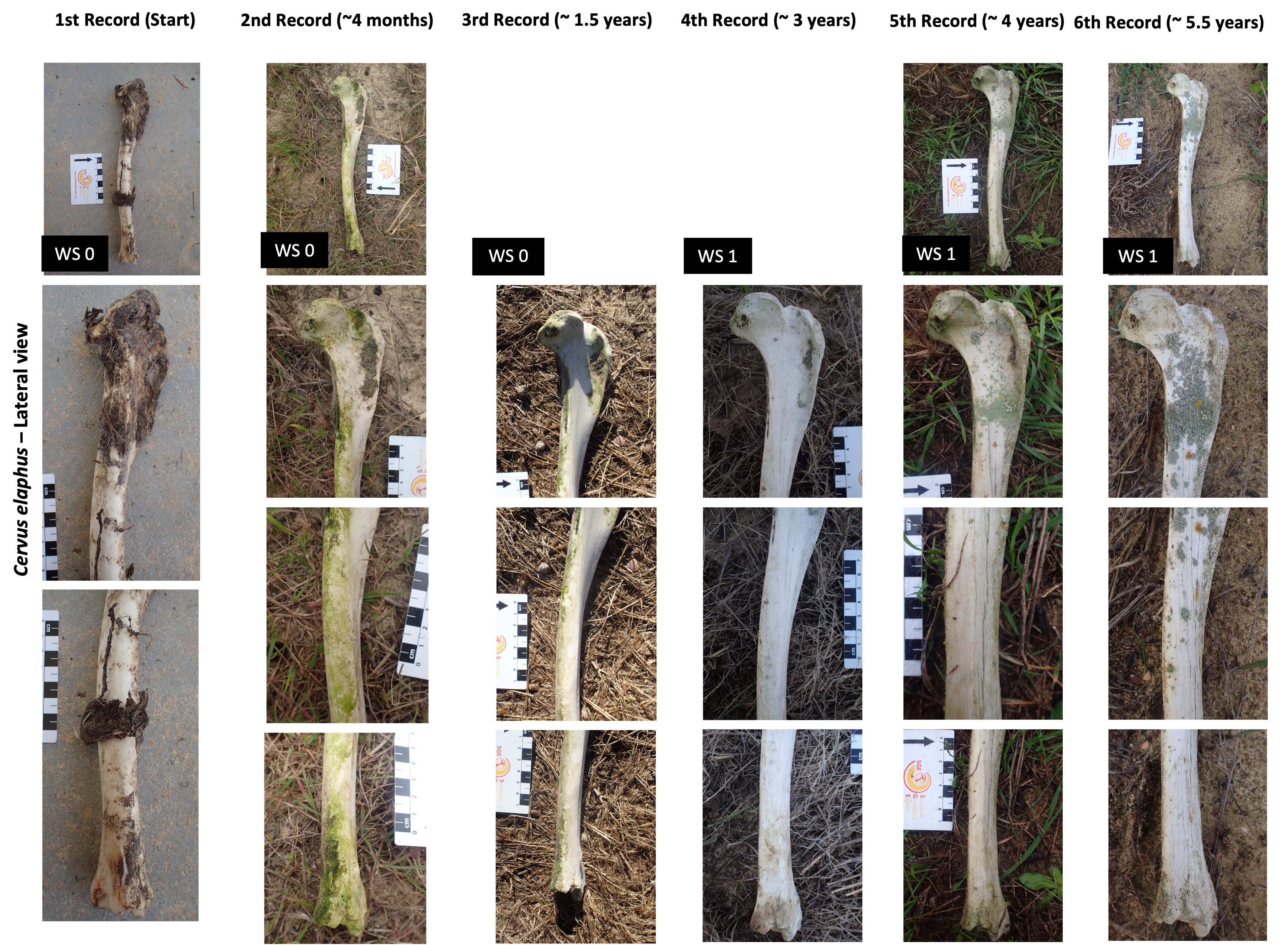

Supplement: S5 Fig — (TIFF) [file pone.0335508.s005.tiff]

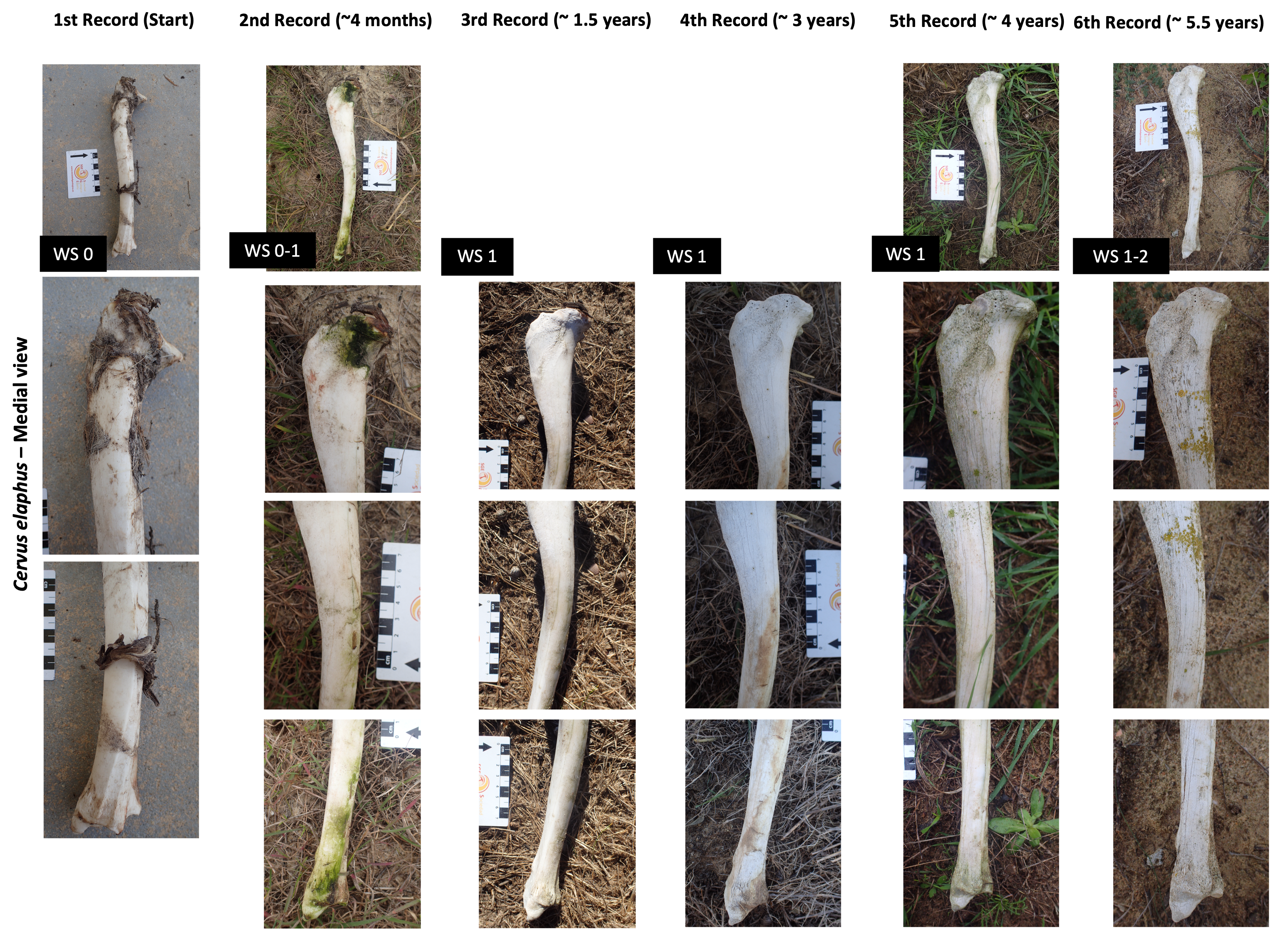

Supplement: S6 Fig — (TIFF) [file pone.0335508.s006.tiff]

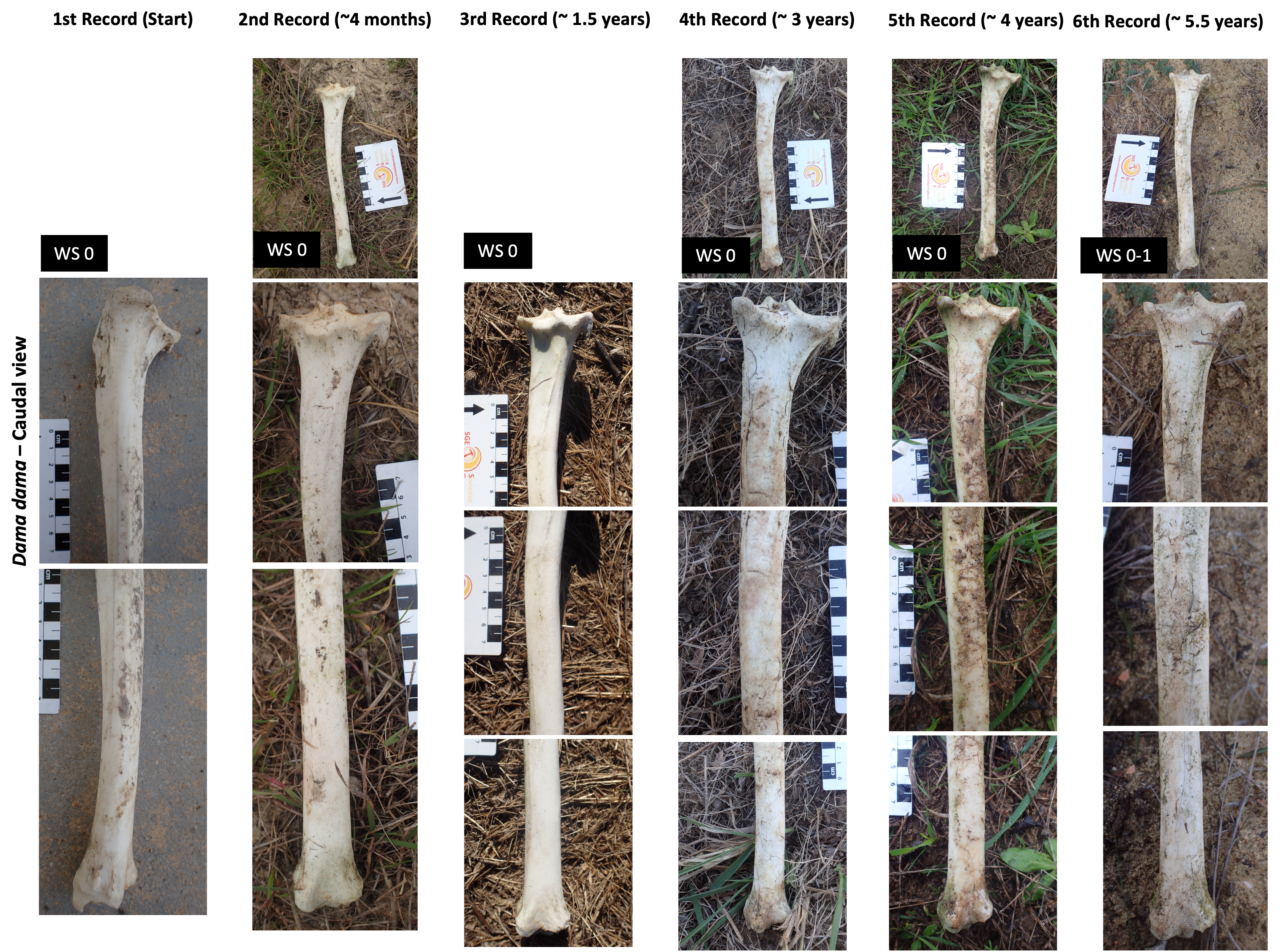

Supplement: S7 Fig — (TIFF) [file pone.0335508.s007.tiff]

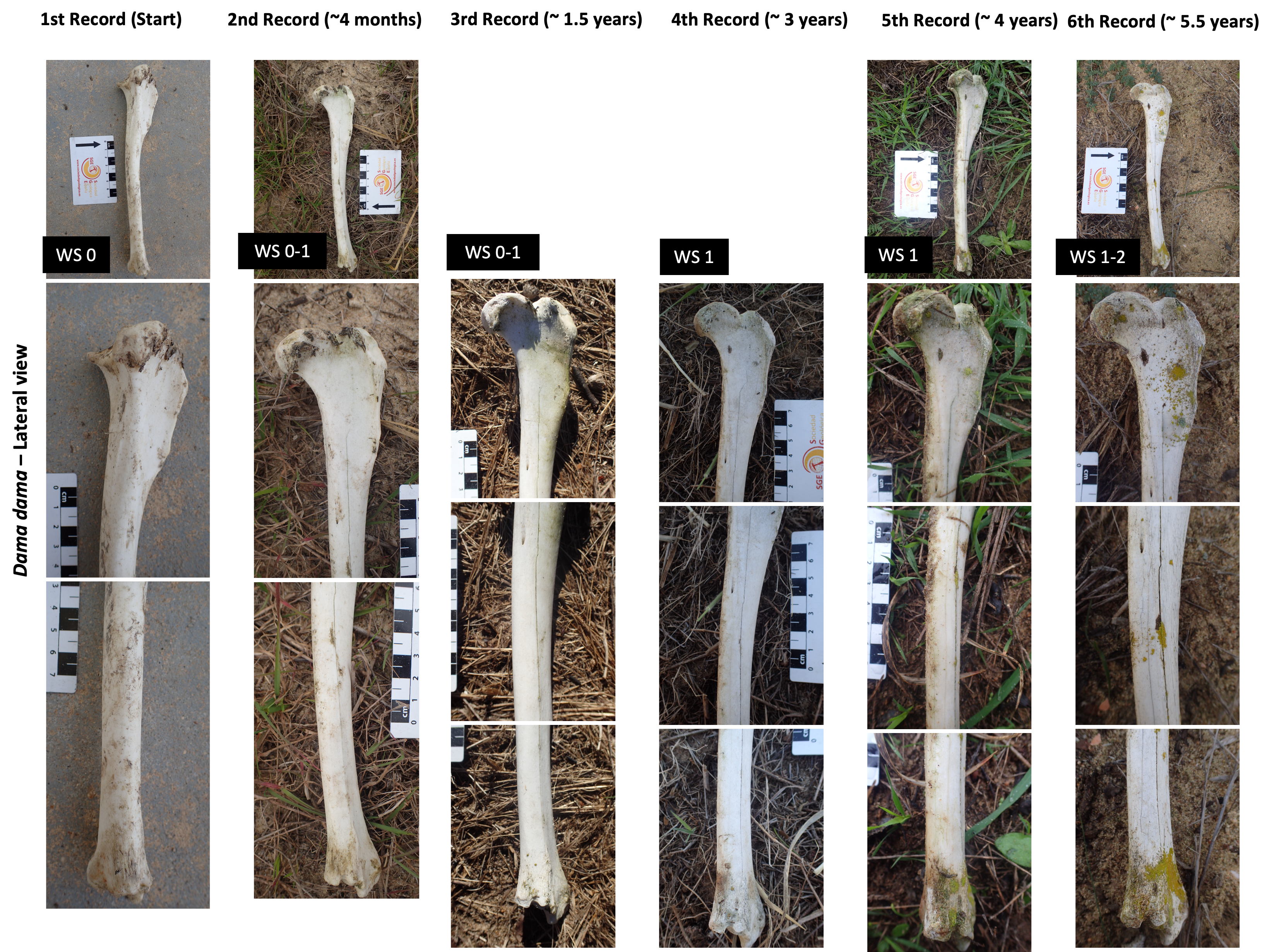

Supplement: S8 Fig — (TIFF) [file pone.0335508.s008.tiff]

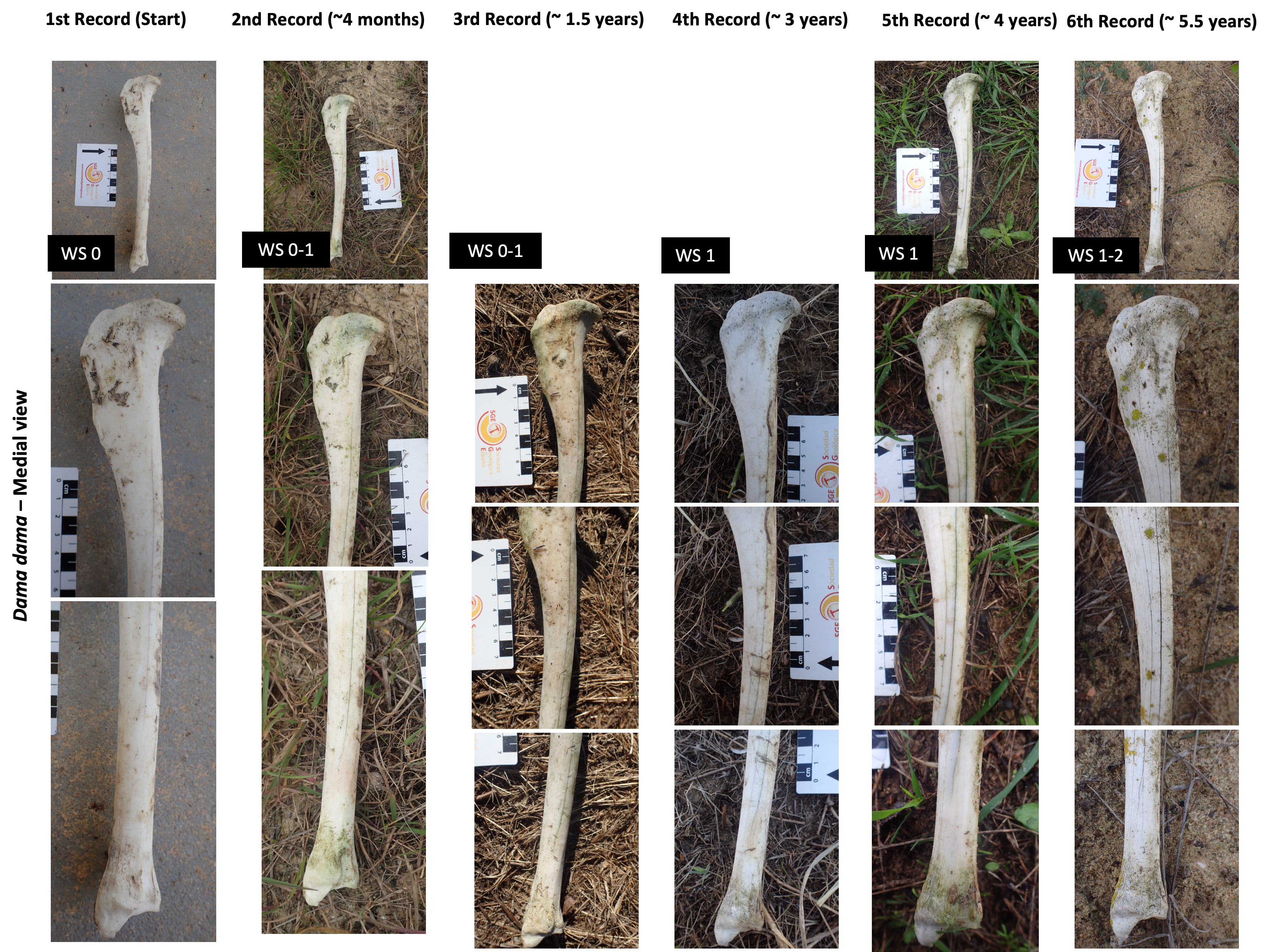

Supplement: S9 Fig — (TIFF) [file pone.0335508.s009.tiff]

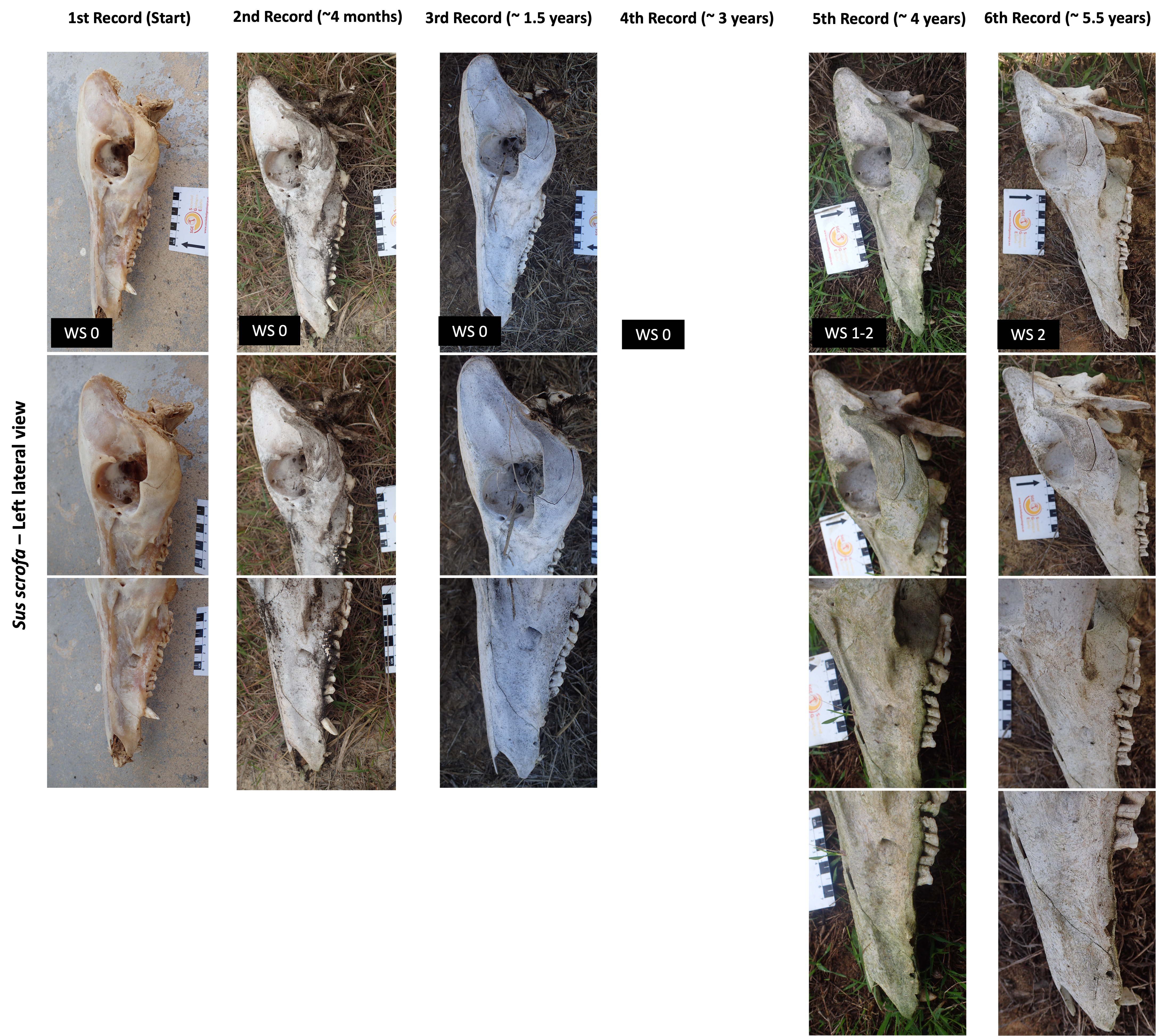

Supplement: S10 Fig — (TIFF) [file pone.0335508.s010.tiff]

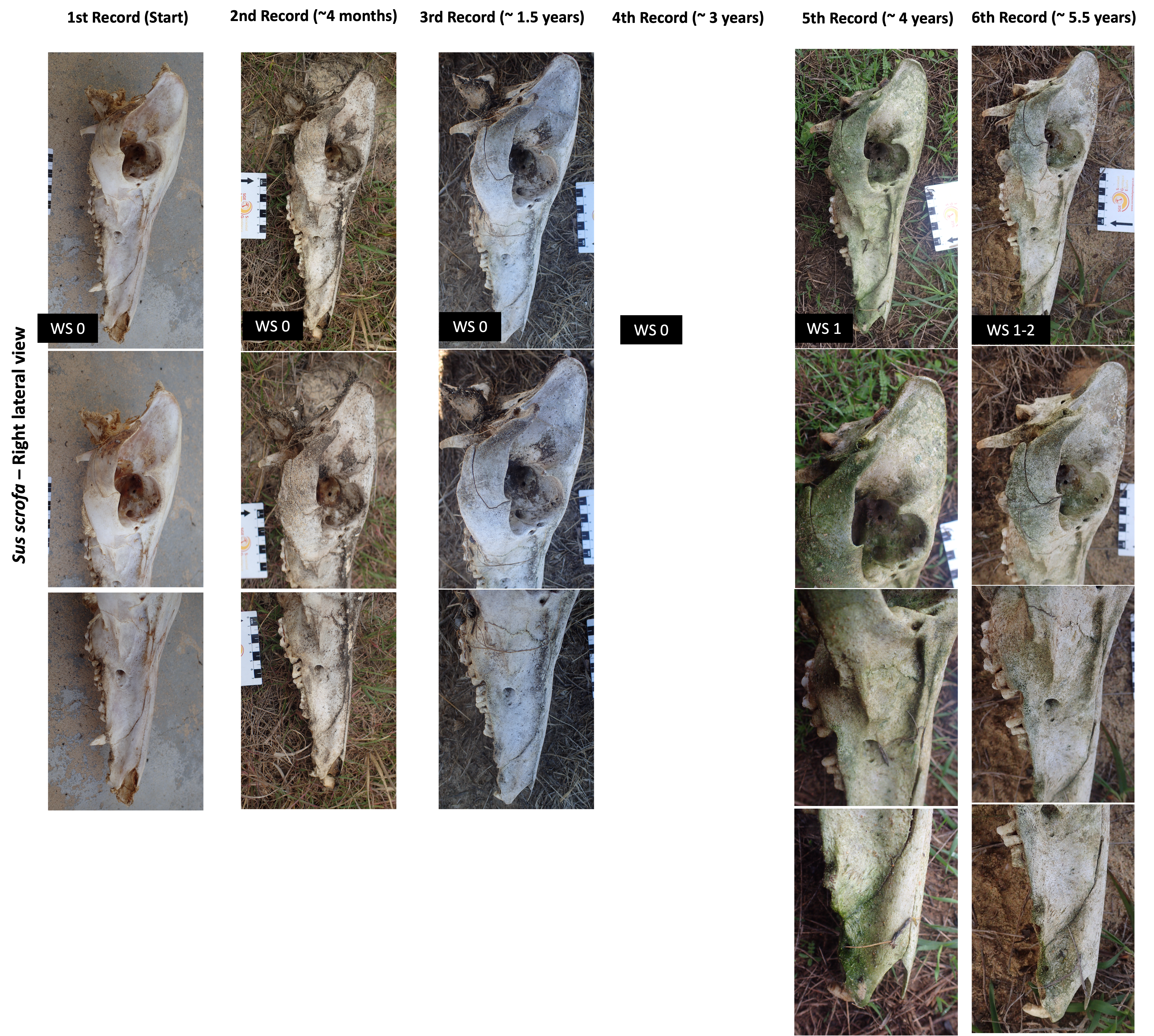

Supplement: S11 Fig — (TIFF) [file pone.0335508.s011.tiff]

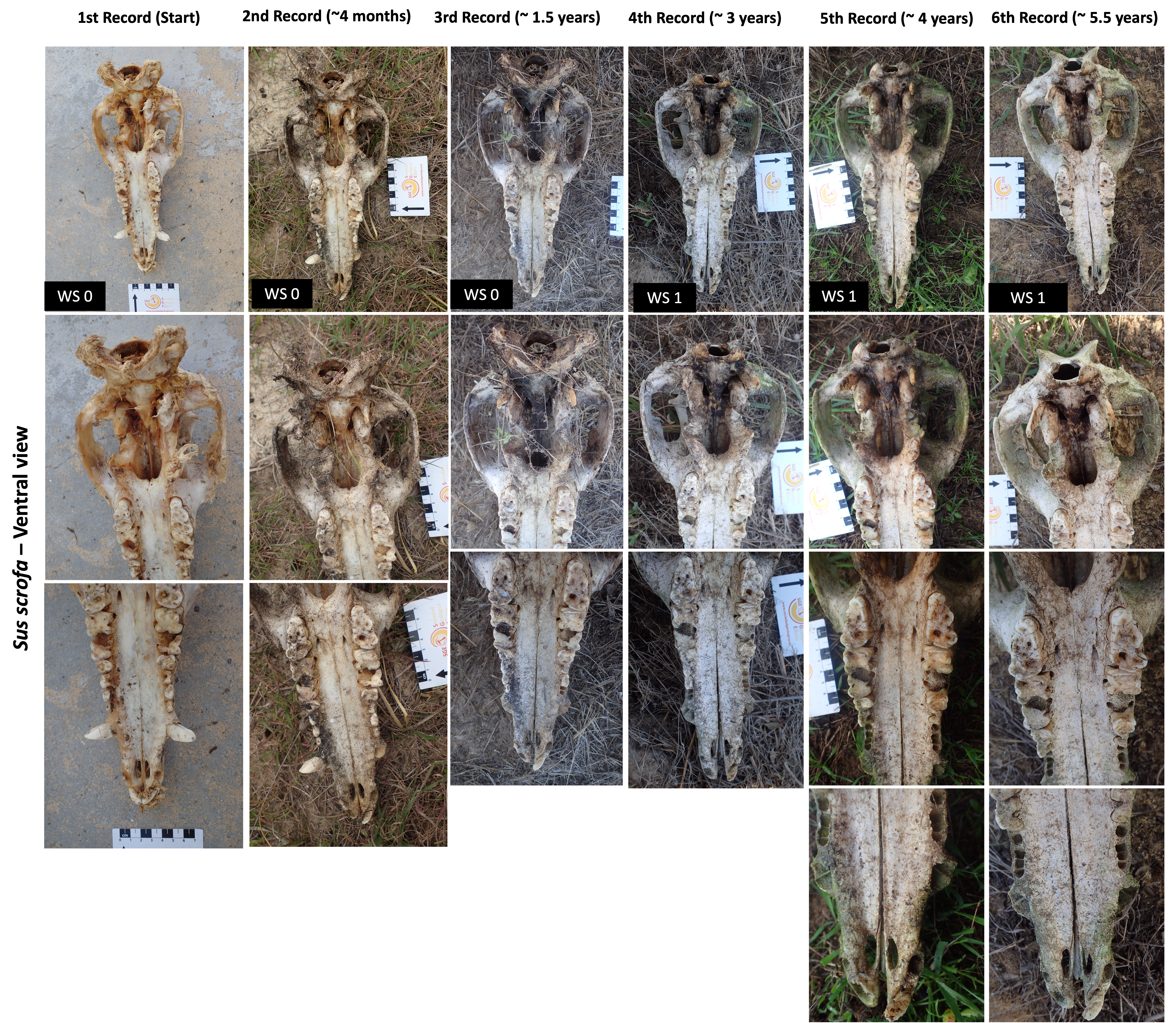

Supplement: S12 Fig — (TIFF) [file pone.0335508.s012.tiff]

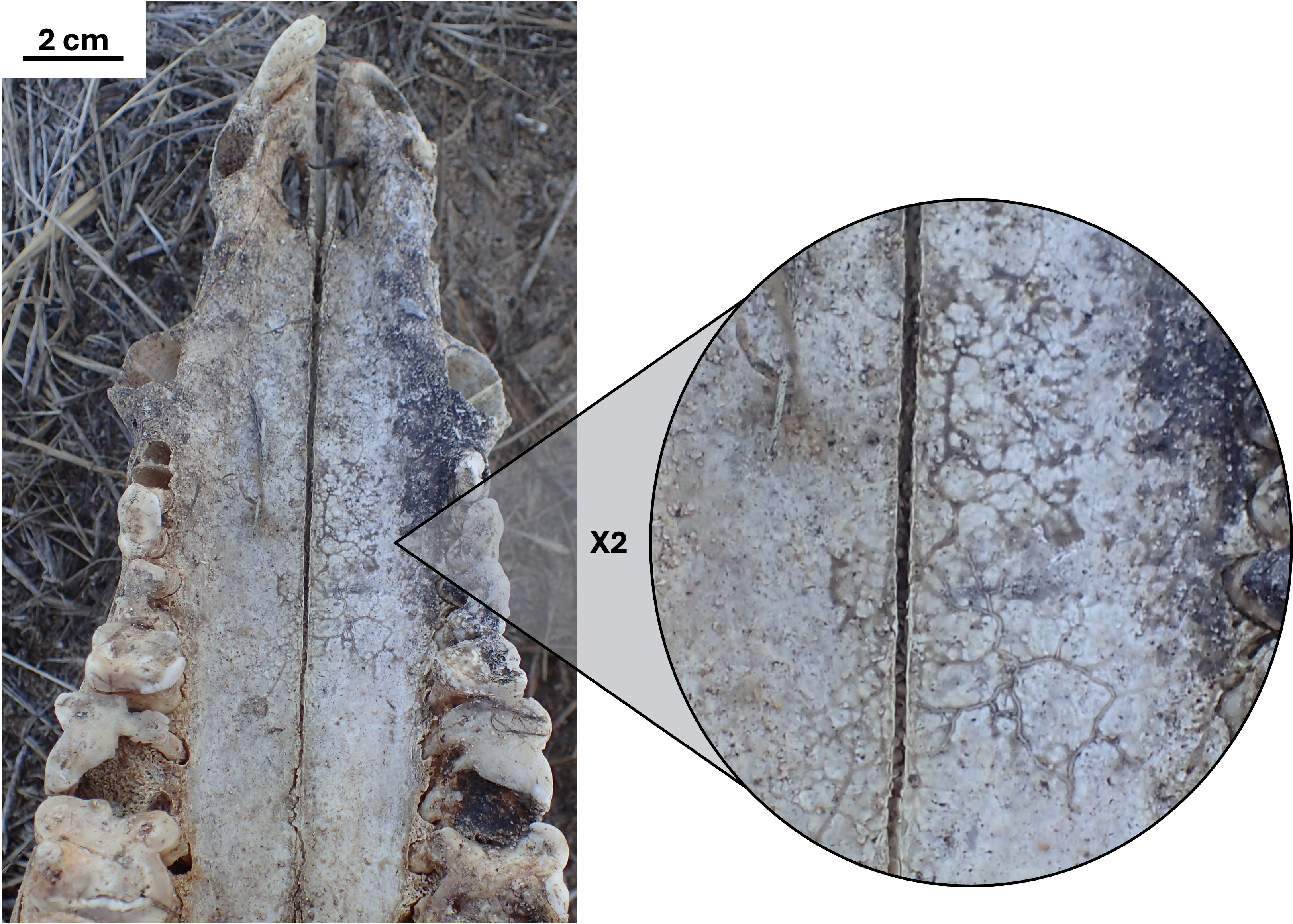

Supplement: S13 Fig — Close-up of the palatal region with fungi hyphae filamentous presence. (TIFF) [file pone.0335508.s013.tiff]
