# Supplementary material for: Bone weathering in a Mediterranean climate region: An experimental case study from Doñana National Park (Spain)
Source: PLoS One. 2025 Oct 31;20(10):e0335508. doi: 10.1371/journal.pone.0335508 (PMC12578198; doi:10.1371/journal.pone.0335508)
Supplement: S1 Table — (PDF) [file pone.0335508.s014.pdf]

	Kruskal-Wallis' test	
	$H(chi^2)$	p(same)
Temperature (ºC)	0.9345	0.9196
Humidity (%)	3.236	0.5192
Rainfall (mm)	1.224	0.8741
Radiation (W/m^2)	4.479	0.345
