# Supplementary material for: Bone weathering in a Mediterranean climate region: An experimental case study from Doñana National Park (Spain)
Source: PLoS One. 2025 Oct 31;20(10):e0335508. doi: 10.1371/journal.pone.0335508 (PMC12578198; doi:10.1371/journal.pone.0335508)
Supplement: S2 Table — (PDF) [file pone.0335508.s015.pdf]

	1st–2nd Record<br>September 18, 2018-<br>January 29, 2019	2nd–3rd Record<br>January 30, 2019-<br>February 3, 2020	3rd–4th Record<br>February 4, 2020-<br>October 19, 2021	4th–5th Record<br>October 20, 2021-<br>December 14, 2022	5th–6th Record<br>December 15, 2022-<br>January 26, 2024
**Temperature 1st-2nd**		0.6106	0.2687	0.4321	0.5473
**Temperature 2nd-3rd**	0.6106		0.2738	0.6468	0.8362
**Temperature 3rd-4th**	0.2687	0.2738		0.4802	0.5114
**Temperature 4th-5th**	0.4321	0.6468	0.4802		0.8103
**Humidity 1st-2nd**		0.4313	0.2979	1	0.4875
**Humidity 2nd-3rd**	0.4313		0.5785	0.3051	0.8722
**Humidity 3rd-4th**	0.2979	0.5785		0.1235	0.7747
**Humidity 4th-5th**	1	0.3051	0.1235		0.3051
**Rainfall 1st-2nd**		0.6106	0.745	0.8614	0.9631
**Rainfall 2nd-3rd**	0.6106		0.3722	0.5557	0.4906
**Rainfall 3rd-4th**	0.745	0.3722		1	0.8531
**Rainfall 4th-5th**	0.8614	0.5557	1		0.8103
**Radiation 1st-2nd**		0.4313	0.07898	0.4321	0.4875
**Radiation 2nd-3rd**	0.4313		0.5557	0.6468	0.7304
**Radiation 3rd-4th**	0.007898	0.5557		0.158	0.1835
**Radiation 4th-5th**	0.4321	0.6468	0.158		0.9826
